# Effects of high CD4 cell counts on death and attrition among HIV patients receiving antiretroviral treatment: an observational cohort study

**DOI:** 10.1038/s41598-017-03384-7

**Published:** 2017-06-09

**Authors:** Zhenzhu Tang, Stephen W. Pan, Yuhua Ruan, Xuanhua Liu, Jinming Su, Qiuying Zhu, Zhiyong Shen, Heng Zhang, Yi Chen, Guanghua Lan, Hui Xing, Lingjie Liao, Yi Feng, Yiming Shao

**Affiliations:** 1 Guangxi Center for Disease Control and Prevention, Nanning, China; 2 Institute for Global Health and Infectious Diseases, University of North Carolina at Chapel Hill, Chapel Hill, USA; 3 State Key Laboratory of Infectious Disease Prevention and Control (SKLID), Collaborative Innovation Center for Diagnosis and Treatment of Infectious Diseases, Chinese Center for Disease Control and Prevention (China CDC), Beijing, China

## Abstract

Current WHO guidelines recommend initiating ART regardless of CD4+ cell count. In response, we conducted an observational cohort study to assess the effects of pre-ART CD4+ cell count levels on death, attrition, and death or attrition in HIV treated patients. This large HIV treatment cohort study (n = 49,155) from 2010 to 2015 was conducted in Guangxi, China. We used a Cox regression model to analyze associations between pre-ART CD4+ cell counts and death, attrition, and death or attrition. The average mortality and ART attrition rates among all treated patients were 2.63 deaths and 5.32 attritions per 100 person-years, respectively. Compared to HIV patients with <350 CD4+ cells/mm^3^ at ART initiation, HIV patients with >500 CD4+ cells/mm^3^ at ART initiation had a significantly lower mortality rate (Adjusted hazard ratio: 0.56, 95% CI: 0.40–0.79), but significantly higher ART attrition rate (AHR: 1.17, 95% CI: 1.03–1.33). Results from this study suggest that HIV patients with high CD4+ cell counts at the time of ART initiation may be at greater risk of treatment attrition. To further reduce ART attrition, it is imperative that patient education and healthcare provider training on ART adherence be enhanced and account for CD4 levels at ART initiation.

## Introduction

Since its development in 1996, highly active antiretroviral therapy (HAART) has been shown to rejuvenate the immune system of HIV infected individuals, prevent onset of opportunistic infections that often lead to death among HIV patients, and generally improve the quality of life among HIV infected persons worldwide^1–4^. Moreover, successful ART has had significant effects on viral suppression and reducing transmission from partner to partner. Recently, a randomized clinical trial and a several observational studies showed that ART can significantly reduce transmissions in serodiscordant heterosexual couples^5–10^. Due to its impact on immunocompetence and HIV transmission, HAART has led to significant declines in AIDS-related morbidity and premature mortality.

Research in recent years has indicated that early initiation of ART can delay time to AIDS-related events and decrease incidences of morbidity and mortality, and high current CD4+ T cell count can be associated with suboptimal adherence to ART^11–17^. Given the clinical benefits and significant reductions in HIV transmission associated with early ART initiation, the World Health Organization (WHO) revised their ART guidelines in 2010 by increasing the threshold for ART eligibility in developed countries from <200 CD4+ cells/ mm^3^ to <350 CD4+ cells/ mm^3^ ^18, 19^. In an effort to end the HIV epidemic by 2030, ART guidelines were again revised in 2015, whereby all people living with HIV were recommended for ART, regardless of WHO clinical stage or CD4 cell count^20^. Implementation of these new guidelines will provide a critical contribution to achieving the UNAIDS’ “90-90-90” target: 90% of all people living with HIV diagnosed, 90% of all people diagnosed with HIV receiving cART, and 90% of all people receiving cART virally suppressed^20, 21^. However, despite recommendations to initiate ART at any CD4 cell count, there exists limited understanding about mortality, attrition, and primary treatment outcomes among HIV patients with ≥500 CD4+ cells/mm^3^over long-durations of ART in real-world settings.

In China, HIV-related mortality has significantly declined since the National Free Antiretroviral Treatment Program (NFATP) was initiated in 2002 and rapidly scaled up^22–26^. Following updated WHO ART guidelines in 2010, China further expanded ART services in order to reduce overall mortality among HIV infected patients, prevent transmission among key populations, and provide ART to those with higher CD4 counts^7, 10, 26^. By the end of 2015, more than 382,139 patients had received ART treatment^27^. Today, China aims to achieve significant reductions in HIV incidence and HIV-related mortality by adopting the UNAIDS 90–90–90 policy.

In Southwest China, the Guangxi Zhuang Autonomous Region accounts for 10% of all reported HIV cases nationwide^28^, and is critical in the country’s HIV prevention and treatment campaign. Situated in the middle of major heroin trafficking routes that begin in Southeast Asia and Southwest China, and continue on towards Guangdong and Hong Kong^29, 30^, injection drug use was the main mode of HIV transmission in the earlier years of the epidemic. However, by the end of 2015, sexual transmission had become the dominant mode of transmission, with heterosexual transmission accounting for 92% of reported HIV cases in Guangxi^28^. Based on a large ART treatment database from Guangxi, we conducted an observational cohort study in order to examine the effects of high CD4+ cell counts (≥500 cells/mm^3^) on treatment outcomes among HIV infected patients receiving ART in a real-world setting.

## Results

### General characteristics of study population

A total of 49309 HIV patients initiated ART between 2010 and 2015 in Guangxi,China, 125 of whom were less than 18 years old, and 29 of whom had no follow-up data. Thus, 49155 eligible study participants were included in this prospective follow-up study. Table 1 presents general characteristics of HIV patients. Patients over 40 years old represented 59.9% of all study participants. The percentage of males was 67.1%, and 68.7% of HIV participants were married. The main route of HIV infection was heterosexual intercourse (88.4%), followed by injection drug use (7.3%), homosexual intercourse (2.1%), and other routes of transmission (2.2%). Prior to ART initiation, patients with CD4+ counts <350 cells/mm^3^, 350–499 cells/mm^3^, and ≥500 cells/mm^3^ accounted for 84.5%, 10.0% and 3.7% of all patients, respectively. Those classified as WHO clinic stage III/IV before ART represented 41.2% of all patients. The percentage of patients who initiated ART with first-line ART and second-line ART was 90.3% and 9.7%, respectively. First-line ART constituted 84.5% of current regimens.

**Table 1:** Characteristics of HIV patients who initiated ART between 2010 and 2015 in Guangxi, China.

Variable	Number	%
Total	49155	100.0
Age (years)		
<40	19728	40.1
≥40	29427	59.9
Sex		
Male	32977	67.1
Female	16178	32.9
Marital status		
Married	33778	68.7
Other	15377	31.3
Route of HIV infection		
Heterosexual intercourse	43441	88.4
Homosexual intercourse	1021	2.1
Injection drug use	3603	7.3
Other	1090	2.2
CD4 count before ART (cells/mm^3^)		
<350	41554	84.5
350–499	4921	10.0
≥500	1796	3.7
Missing	884	1.8
WHO clinic stage before ART		
I/II	28919	58.8
III/IV	20236	41.2
Initial ART regimen		
The first-line ART	44388	90.3
The second-line ART	4767	9.7
Current ART regimen		
The first-line ART	41558	84.5
The second-line ART	7597	15.5
Year of ART initiation		
2010	5710	11.6
2011	7743	15.8
2012	9210	18.7
2013	8850	18.0
2014	9440	19.2
2015	8202	16.7

### Mortality rates

Among all eligible HIV infected patients who initiated ART between 2010 and 2015 in Guangxi, China, 3411 deaths were observed, 4507 patients lost to follow-up, and 2396 patients stopped ART. Of the 2396 patients who stopped ART, 1358 (56.7%) were due to poor adherence, 437 (18.2%) were due to drug side effects, 70 (2.9%) were due to economic issues, and 531(22.2%) were due to other reasons. The average mortality rate was 2.63 deaths per 100 person-years among all patients (Table 2).

**Table 2:** Mortality rates in HIV-infected patients who started ART between 2010 and 2015 in Guangxi, China, by year post-ART initiation.

Variable	Number of HIV patients	Deaths	Person years	Deaths/100 person year (95% CI)
Overall	49155	3411	129837.65	2.63(2.54–2.72)
0 to 1 year	49155	2066	44361.04	4.66(4.46–4.86)
0 to 2 year	49155	2719	77506.09	3.51(3.38–3.64)
0 to 3 year	49155	3080	101464.68	3.04(2.93–3.14)
0 to 4 year	49155	3298	117556.62	2.81(2.71–2.90)
0 to 5 year	49155	3382	126356.97	2.68(2.59–2.77)
0 to 6 year	49155	3411	129837.65	2.63(2.54–2.72)

### Attrition rates

Among all eligible HIV infected patients who initiated ART between 2010 and 2015 in Guangxi, China, 6903 attritions were observed. Of 6903 patient attritions, 4507 patients were lost to follow-up, and 2396 patients stopped ART. The average attrition rate was 5.32 attritions per 100 person-years among all treated patients (Table 3).

**Table 3:** Attrition rates in HIV-infected patients who started ART between 2010 and 2015 in Guangxi, China, by year post-ART initiation.

Variable	Number of HIV patients	Attritions	Person years	Attritions/100 person year (95% CI)
Overall	49155	6903	129837.65	5.32(5.19–5.44)
0 to 1 year	49155	4178	44361.04	9.42(9.13–9.70)
0 to 2 year	49155	5515	77506.09	7.12(6.93–7.30)
0 to 3 year	49155	6244	101464.68	6.15(6.00–6.31)
0 to 4 year	49155	6612	117556.62	5.62(5.49–5.76)
0 to 5 year	49155	6823	126356.97	5.40(5.27–5.53)
0 to 6 year	49155	6903	129837.65	5.32(5.19–5.44)

### Death + attrition rates

Among all eligible HIV infected patients who initiated ART between 2010 and 2015 in Guangxi, China, 10314 death + attritions were observed. The average death + attrition rate was 7.94 per 100 person-years among all treated patients (Table 4).

**Table 4:** Dearth + attrition rates in HIV-infected patients who started ART between 2010 and 2015 in Guangxi, China, by year post-ART initiation.

Variable	Number of HIV patients	Death + attritions	Person years	Death + attritions/100 person year (95% CI)
Overall	49155	10314	129837.65	7.94(7.79–8.10)
0 to 1 year	49155	6244	44361.04	14.08(13.73–14.42)
0 to 2 year	49155	8234	77506.09	10.62(10.39–10.85)
0 to 3 year	49155	9324	101464.68	9.19(9.00–9.38)
0 to 4 year	49155	9910	117556.62	8.43(8.26–8.60)
0 to 5 year	49155	10205	126356.97	8.08(7.92–8.23)
0 to 6 year	49155	10314	129837.65	7.94(7.79–8.10)

### Effects of pre-ART CD4 count on death

Table 5 presents the unadjusted and adjusted effects of pre-ART CD4 counts on death after ART initiation. In the adjusted models, 350–499 CD4 cells/mm^3^ before ART (compared to <350 cells/mm^3^ before ART, AHR = 0.45, 95% CI: 0.36–0.56) and ≥500 CD4 cells/mm^3^ before ART (compared to <350 cells/mm^3^ before ART, AHR = 0.56, 95% CI: 0.40–0.79) were both inversely significantly associated with death in HIV patients who started ART. Similar effects of pre-ART CD4 counts on death after ART initiation were observed even after excluding individuals with missing baseline CD4 counts (Supplementary Table 2).

**Table 5:** Effects of CD4 count before ART on death in HIV-infected patients who started ART between 2010 and 2015 in Guangxi, China.

Variable	Number	Deaths	Person years	Deaths/100 person years (95% CI)	HR (95% CI)	P-value	AHR^*^ (95% CI)	P-value
Total	49155	3411	129837.65	2.63(2.54–2.72)				
CD4 count before ART (cells/mm^3^)								
<350	41554	3208	114372.74	2.80(2.71–2.90)	1.00		1.00	
350–499	4921	86	10118.95	0.85(0.67–1.03)	0.26(0.21–0.32)	<0.01	0.45(0.36–0.56)	<0.01
≥500	1796	36	3328.63	1.08(0.73–1.43)	0.31(0.22–0.43)	<0.01	0.56(0.40–0.79)	0.001
Missing	884	81	2017.31	4.02(3.14–4.89)	1.31(1.05–1.64)	0.016	1.57(1.26–1.95)	<0.01

^*^HR = hazard ratio; AHR = adjusted hazard ratio; covariates of the adjusted model included: age, sex, marital status, route of HIV infection, WHO clinic stage before ART, initial ART regimen, year initiated ART.

### Effects of pre-ART CD4 count on attrition

Table 6 presents the unadjusted and adjusted effects of pre-ART CD4+ count on attrition after ART initiation. Compared to having <350 CD4 cells/mm^3^ pre-ART initiation, having 350–499 CD4 cells/mm^3^ at pre-ART initiation was marginally significantly associated with attrition (adjusted hazard ratio (AHR = 1.08, 95% CI: 0.99–1.17), and having ≥500 CD4 cells/mm^3^ at pre-ART initiation was significantly associated with attrition (AHR = 1.17, 95% CI: 1.03–1.33). Similar effects of pre-ART CD4 counts on attrition after ART initiation were observed even after excluding individuals with missing baseline CD4 counts (Supplementary Table 3).

**Table 6:** Effects of CD4 count before ART on attrition in HIV-infected patients who started ART between 2010 and 2015 in Guangxi, China.

Variable	Number	Attritions	Person years	Attritions/100 person years (95% CI)	HR (95% CI)	P-value	AHR^*^ (95% CI)	P-value
Total	49155	6903	129837.65	5.32(5.19–5.44)				
CD4 count before ART (cells/mm^3^)								
<350	41554	5803	114372.74	5.07(4.94–5.20)	1.00		1.00	
350–499	4921	660	10118.95	6.52(6.02–7.02)	1.10(1.02–1.20)	0.016	1.08(0.99–1.17)	0.081
≥500	1796	254	3328.63	7.63(6.69–8.57)	1.23(1.08–1.39)	0.001	1.17(1.03–1.33)	0.019
Missing	884	186	2017.31	9.22(7.90–10.55)	1.68(1.46–1.95)	<0.01	1.61(1.39–1.86)	<0.01

^*^HR = hazard ratio; AHR = adjusted hazard ratio; covariates of the adjusted model included: age, sex, marital status, route of HIV infection, WHO clinic stage before ART, initial ART regimen, year initiated ART.

### Effects of pre-ART CD4 count on death + attrition

Table 7 presents the unadjusted and adjusted effects of pre-ART CD4+ count on death + attrition after ART initiation. Compared to having <350 CD4+ cells/mm^3^ pre-ART initiation, neither having 350–499 CD4+ cells/mm^3^ at pre-ART initiation nor having ≥ 500 CD4+ cells/mm^3^ at pre-ART initiation was significantly associated with death + attrition (AHR = 0.94, 95% CI: 0.87–1.01 and AHR = 1.04, 95% CI: 0.93–1.18, respectively). Similar effects of pre-ART CD4 counts on death + attrition after ART initiation were observed even after excluding individuals with missing baseline CD4 counts (Supplementary Table 4).

**Table 7:** Effects of CD4 count before ART on Death + attritions in HIV-infected patients who started ART between 2010 and 2015 in Guangxi, China.

Variable	Number	Death + attritions	Person years	Death + attritions /100 person years (95% CI)	HR (95% CI)	P-value	AHR^*^ (95% CI)	P-value
Total	49155	10314	129837.65	7.94(7.79–8.10)				
CD4 count before ART (cells/mm^3^)								
<350	41554	9011	114372.74	7.88(7.72–8.04)	1.00		1.00	
350–499	4921	746	10118.95	7.37(6.84–7.90)	0.80(0.74–0.86)	<0.01	0.94(0.87–1.01)	0.105
≥500	1796	290	3328.63	8.71(7.71–9.72)	0.90(0.80–1.01)	0.077	1.04(0.93–1.18)	0.478
Missing	884	267	2017.31	13.24(11.65–14.82)	1.55(1.37–1.75)	<0.01	1.60(1.41–1.81)	<0.01

^*^HR = hazard ratio; AHR = adjusted hazard ratio; covariates of the adjusted model included: age, sex, marital status, route of HIV infection, WHO clinic stage before ART, initial ART regimen, year initiated ART.

### Effects of CD4 count before ART on loss to follow-up and medication cessation

We conducted a separate analysis for loss to follow-up and medication cessation as the outcomes of interest (Supplementary Tables 6–9). Generally, the effect of ≥500 CD4 cells/mm^3^ at pre-ART baseline on attrition was similar to its effects on loss to follow-up and medication cessation.

## Discussion

Although the “90-90-90” targets and corresponding HIV treatment guidelines have already been endorsed by UNAIDS^20^, additional high quality evidence is needed to better understand death and attrition rates among those who initiate ART with pre-ART ≥500 CD4+ cells/mm^3^. However, most relevant studies that have examined patient death and treatment attrition after ART initiation, have defined early treatment as initiating ART when CD4+ counts were ≥350 (cells/mm^3^)^11–13, 15–17^. To better understand the effects of high CD4+ cell counts (≥500 cells/mm^3^) on death and treatment attrition among HIV infected patients receiving ART in a real-world setting, we conducted a large observational cohort study among 49309 HIV+ patients receiving ART in rural Southwest China.

Our study found that the overall mortality rate was 2.63 per 100 person-years in HIV infected patients who started ART between 2010 and 2015 in Guangxi, China. Despite the achievements of China’s NFATP, this mortality rate remains much higher than that in developed countries^2, 3^. Continued improvement in the quality of HIV treatment and care is needed to further reduce the mortality rate in Chinese patients.

This large observational cohort study also showed that higher pre-ART CD4 cell counts (350–499 CD4+ cells/mm^3^ and >500 CD4+ cells/mm^3^ versus <350 cells/mm^3^) were associated with lower mortality risk, confirming the results of previous studies^11–13, 15–17^. Such findings lend strong support to China’s adoption of the UNAIDs “90–90–90” policy, which includes continued expansion of HIV counseling and testing, and ART uptake by 90% of all people with diagnosed HIV infection^20, 21^.

The overall attrition rate among HIV patients in this study was 5.32 per 100 person-years, which is lower than that in other developing countries, but higher than that in developed countries^31, 32^. Compared with pre-ART CD4 counts <350 cells/mm^3^, attrition was marginally significantly associated with pre-ART CD4 counts 350–499 cells/mm^3^, and significantly associated with pre-ART CD4 counts >500 cells/mm^3^. One major concern with initiating cART among patients with a high CD4+ cell counts is increased risk of poor adherence. Given that patients with higher CD4+ cell counts are more likely to be AIDS asymptomatic, such individuals may be less willing or motivated to take cART^33–36^. High current CD4+ counts was strongly associated with lower adherence to ART, thus indicating that enhancement of adherence education is needed, especially for patients with high CD4+ counts^17^. In addition, attritions were associated with both individual and contextual factors, such as younger age, male gender, being single or divorced, poor adherence and drug side effects^33^. Given that retention in ART and care can help improve individual clinical outcomes, evaluating and optimizing HIV clinical care is essential for developing effective adherence interventions to reduce attrition. In China, TDF has replaced both stavudine (D4T) and zidovudine (AZT) given its more favorable toxicity profile, dosing, and cost-effectiveness^37^. It is imperative for China to identify suitable timing to implement policies recommending initiation of ART regardless of CD4 cell counts. Rates of both death and attrition were highest among participants with missing CD4 count data, possibly due to a high proportion of individuals with WHO clinic stage III/IV before ART (31%) and possibly poor linkage to care. This study is arguably the first large, empirical study to suggest negative population-level consequences of initiating ART among HIV patients with pre-ART CD4+ counts ≥500 cells/mm^3^.

Our study has several limitations which warrant noting. First, although we controlled for numerous potential confounders in multivariable modeling, selection bias in the observational cohort study may have been an issue. As the study did not include individuals who were diagnosed with HIV but did not initiate ART, it is possible that individuals with poorer health-seeking behaviors were underrepresented in the study. Second, the median duration of follow-up was not very long. As time allows for longer-term studies of the new WHO treatment guidelines, future studies should consider using marginal structural models to evaluate the long-term effects of pre-ART CD4+ counts on treatment outcomes among HIV-infected patients receiving ART. Third, our study used all-cause mortality rather than HIV-related death, which arguably would have been more accurate for evaluating treatment effects. Fourth, this study was conducted in Guangxi, which only represents 13% of all HIV patients on ART in China^28^. Findings might not be fully representative of China. Fifth, parameter estimates may have been partially biased by the presence of unobserved time-varying confounders. Sixth, because lost to follow-up may have been associated with earlier time to death, mortality rate estimates may have been lower than the true rate.

To our knowledge, this is the first large-scale observational cohort study in a developing country to evaluate the effects of high CD4+ cell counts on treatment outcomes among HIV infected patients receiving ART. Novel results from this study provide compelling evidence for policy-makers to prioritize ART healthcare provider training, ART patient education, and the availability of simpler, more effective, safer and better tolerated regimens, especially when initiating ART among HIV-infected patients with high CD4+ cell counts. Currently, China’s HIV treatment policies reflect the WHO and UNAIDS recommended guidelines to initiate treatment among all HIV patients regardless of CD4+ count, but further monitoring of treatment outcomes is needed to elucidate the determinants of long-term programmatic success.

## Methods

### Study design and study participants

This HIV ART observational cohort study was conducted in Guangxi, rural southwest China. Patients initiating free ART through the NFATP between January 1, 2010 and October 31, 2015 were included. Patients who did not initiate ART during that time frame were not included in the study. Individuals who initiated free ART and were at least 18 years old at the time of ART initiation and provided informed consent were eligible to participate in the study. The Chinese national ART treatment criteria since 2010 was: (1) CD4 cell count <350/mm^3^; or (2) World Health Organization (WHO) stage III/IV clinic conditions; or (3) willingness to receive ART, regardless of the criteria 1 and 2, such as initiating ART at high CD4+ cell counts^37^. Currently, first-line ART regimens consist of [tenofovir (TDF) or azidothymidine (AZT)] + lamivudine (3TC) + [efavirenz (EFV) or nevirapine (NVP)]. Second-line ART regimens consist of TDF + 3TC + EFV/(LPV/r). By the end of 2015, more than 62,967 HIV patients had received ART in Guangxi, representing 13% of the total number of HIV patients on treatment in China^28^. This study and all methods were approved by the institutional review board (IRB) of the Guangxi Center for Disease Control and Prevention. All research methods in this study were carried out in accordance with the approved guidelines.

### Data collection

Variables collected at baseline enrollment into the HIV ART observational cohort included: age, sex, ethnicity, marital status, route of HIV infection, CD4 count in the six months before ART, WHO clinic stage in the six months before ART, initial ART regimen, current ART regimen, and year of ART initiation. Variables collected at each follow-up included: transferals to another clinic, cessation of ART, loss to follow-up, duration of ART, and survival status. The follow up visits occurred at 0.5, 1, 2, and 3 months following ART initiation, and then every 3 months thereafter. Lost to follow-up was defined as missing more than 90 days after the last date seen in clinic, which was also defined as the date of withdrawal.

### Statistical analysis

We conducted a prospective cohort analysis using Guangxi data from the NFATP. Treatment outcomes included death and attrition. Attrition was defined as cessation of ART or lost to follow-up as reported through the NFATP database. Time zero was defined as the date of ART initiation, and data were censored at April 30, 2016. Incidence rates were calculated based on Poisson distributions. Death, attrition, and death or attrition (death + attrition) incidence rates and their 95% confidence intervals (CI) were analyzed with incidence densities per 100 person-years of follow-up.

Cause-specific Cox proportional hazard models^38^ were used to evaluate effects of CD4+ count levels before ART on death, attrition, and death + attrition. Competing risks for cause-specific hazard models were censored accordingly. In order to control for potential confounding, the following baseline covariates of the adjusted model were included: age, sex, ethnicity, marital status, route of HIV infection, WHO clinic stage before ART, initial ART regimen, and year initiated ART. A two-sided p-value of 0.05 or less was regarded as statistically significant. Statistical Analysis System (SAS 9.1^TM^ for Windows; SAS Institute Inc., NC, USA) was used for all the analyses.

### Management of missing baseline CD4 data

A sensitivity analysis was conducted to assess for potential bias resulting from the 1.8% of individuals with missing baseline CD4 data, the only covariate with any missing data. First, we compared baseline demographics and treatment variables for complete cases (n = 48,271) and cases with missing baseline CD4 data (n = 884) (Supplementary Table 1). Results showed that demographics were similar between complete and missing cases, although cases with missing CD4 data appeared slightly less likely to be diagnosed as WHO clinic stage III/IV, compared to complete cases overall (31.4% vs 41.3%). Then, we re-analyzed the data using a complete-case analysis approach, whereby individuals with missing CD4 data were excluded from analysis (Supplementary Tables 2–4). Results of the sensitivity analysis indicated that effects of pre-ART CD4 counts on death, attrition, and death + attrition, were not meaningfully influenced by inclusion or exclusion of individuals with missing baseline CD4 data.

We also examined the proportion of individuals diagnosed as WHO clinic stage III/IV, stratified by baseline CD4 count in order to draw inferences about baseline CD4 counts among individuals with missing baseline CD4 data (Supplementary Table 5). Results showed that individuals with missing baseline CD4 data were four times more likely to be diagnosed as WHO clinic stage III/IV, compared to individuals with baseline CD4 counts greater than 500 cells/mm^3^ (31.5% vs 8.1%). Given that 46.8% of individuals with baseline CD4 counts <350 cells/mm^3^ were diagnosed as WHO clinic stage II/IV and assuming moderate to strong correlation between WHO clinical staging and baseline CD4 counts, these results suggest that the majority of individuals with missing baseline CD4 counts had actual CD4 counts below 350 cells/mm^3^.

## Electronic supplementary material

supplementary tables
