# Supplementary material for: Effects of high CD4 cell counts on death and attrition among HIV patients receiving antiretroviral treatment: an observational cohort study
Source: Sci Rep. 2017 Jun 9;7:3129. doi: 10.1038/s41598-017-03384-7 (PMC5466653; doi:10.1038/s41598-017-03384-7)
Supplement: Supplementary file 1 — supplementary tables [file 41598_2017_3384_MOESM1_ESM.pdf]

Effects of high CD4 cell counts on death and attrition among HIV patients receiving antiretroviral treatment: an observational cohort study

Zhenzhu Tang[1], Stephen W. Pan[2], Yuhua Ruan[1,3], Xuanhua Liu[1], Jinming Su[1], Qiuying Zhu[1], Zhiyong Shen[1], Heng Zhang[3], Yi Chen[1], Guanghua Lan[1], Hui Xing[3], Lingjie Liao[3], Yi Feng[3], Yiming Shao[1,3]

1. Guangxi Center for Disease Control and Prevention, Nanning, China
2. Institute for Global Health and Infectious Diseases, University of North Carolina at Chapel Hill, Chapel Hill, USA
3. State Key Laboratory of Infectious Disease Prevention and Control (SKLID), Collaborative Innovation Center for Diagnosis and Treatment of Infectious Diseases, Chinese Center for Disease Control and Prevention (China CDC), Beijing, China

Correspondence:

Yiming Shao, MD., Ph.D.

State Key Laboratory of Infectious Disease Prevention and Control (SKLID), Collaborative Innovation Center for Diagnosis and Treatment of Infectious Diseases, Chinese Center for Disease Control and Prevention (China CDC), Beijing, China

155 Changbai Road, Changping District, Beijing, 102206, P. R. China

Telephone: 86 10 58900981; Fax: 86 10 58900981

E-mail: yshao@bjmu.edu.cn

Supplementary Table 1: Baseline measures among complete cases and cases with missing CD4 data

**Variable**	**Complete cases (%)**	**Missing CD4 data (%)**
Overall	48271(98.2)	884(1.8)
Age (years)		
<40	19330(40.0)	398(45.0)
≥40	28941(60.0)	486(55.0)
Sex		
Male	32368(67.1)	609(68.9)
Female	15903(32.9)	275(31.1)
Marital status		
Married	33187(68.8)	591(66.9)
Other	15084(31.2)	293(33.1)
Route of HIV infection		
Heterosexual intercourse	42693(88.4)	748(84.6)
Homosexual intercourse	1003(2.1)	18(2.0)
Intravenous drug use	3521(7.3)	82(9.3)
Other	1054(2.2)	36(4.1)
WHO clinic stage before ART		
I/II	28313(58.7)	606(68.6)
III/IV	19958(41.3)	278(31.4)
Initial ART regiment		
The first-line ART	43628(90.4)	760(86.0)
The second-line ART	4643(9.6)	124(14.0)
Current ART regiment		
The first-line ART	40812(84.5)	746(84.4)
The second-line ART	7459(15.5)	138(15.6)
Year of ART initiation		
2010	5666(11.7)	44(5.0)
2011	7602(15.7)	141(16.0)
2012	9058(18.8)	152(17.2)
2013	8622(17.9)	228(25.8)
2014	9259(19.2)	181(20.5)
2015	8064(16.7)	138(15.6)

Supplementary Table 2. Effects of CD4 count before ART on death in HIV-infected patients who started ART between 2010 and 2015 in Guangxi, China (Analytic sample does not include individuals with missing baseline CD4 counts)

Variable	Number	Deaths	Person years	Deaths/100 person years (95% CI)	HR (95% CI)	P-value	AHR* (95% CI)	P-value
Total	48271	3330	127820.33	2.61(2.52-2.69)				
CD4 count before ART (cells/mm^3)								
<350	41554	3208	114372.74	2.80(2.71-2.90)	1.00		1.00	
350-499	4921	86	10118.95	0.85(0.67-1.03)	0.26(0.21-0.32)	<0.01	0.45(0.36-0.55)	<0.01
≥500	1796	36	3328.63	1.08(0.73-1.43)	0.31(0.22-0.43)	<0.01	0.56(0.40-0.78)	0.001

* HR=hazard ratio; AHR=adjusted hazard ratio; covariates of the adjusted model included: age, sex, marital status, route of HIV infection, WHO clinic stage before ART, initial ART regiment, year initiated ART.

Supplementary Table 3. Effects of CD4 count before ART on attrition in HIV-infected patients who started ART between 2010 and 2015 in Guangxi, China (Analytic sample does not include individuals with missing baseline CD4 counts)

Variable	Number	attritions	Person years	Attritions/100 person years (95% CI)	HR (95% CI)	P-value	AHR* (95% CI)	P-value
Total	48271	6717	127820.33	5.26(5.13-5.38)				
CD4 count before ART (cells/mm^3)								
<350	41554	5803	114372.74	5.07(4.94-5.20)	1.00		1.00	
350-499	4921	660	10118.95	6.52(6.02-7.02)	1.10(1.02-1.20)	0.016	1.08(0.99-1.17)	0.082
≥500	1796	254	3328.63	7.63(6.69-8.57)	1.23(1.08-1.39)	0.001	1.17(1.03-1.32)	0.019

* HR=hazard ratio; AHR=adjusted hazard ratio; covariates of the adjusted model included: age, sex, marital status, route of HIV infection, WHO clinic stage before ART, initial ART regiment, year initiated ART.

Supplementary Table 4. Effects of CD4 count before ART on Death + attritions in HIV-infected patients who started ART between 2010 and 2015 in Guangxi, China (Analytic sample does not include individuals with missing baseline CD4 counts)

Variable	Number	Death + attritions	Person years	Death + attritions /100 person years (95% CI)	HR (95%CI)	P-value	AHR* (95%CI)	P-value
Total	48271	10047	127820.33	7.86(7.71-8.01)				
CD4 count before ART (cells/mm^3)								
<350	41554	9011	114372.74	7.88(7.72-8.04)	1.00		1.00	
350-499	4921	746	10118.95	7.37(6.84-7.90)	0.80(0.74-0.86)	<0.01	0.94(0.87-1.02)	0.118
≥500	1796	290	3328.63	8.71(7.71-9.72)	0.90(0.80-1.01)	0.076	1.05(0.93-1.18)	0.458

* HR=hazard ratio; AHR=adjusted hazard ratio; covariates of the adjusted model included: age, sex, marital status, route of HIV infection, WHO clinic stage before ART, initial ART regiment, year initiated ART.

Supplementary Table 5: WHO clinic stage III/IV by baseline CD4 count

Variable	Total	WHO clinic stage III/IV before ART (%)		
Overall	49155	20236 (41.2)		
CD4 count before ART (cells/mm^3)				
<350	41554	19430 (46.8)		
350-499	4921	383 (7.8)		
≥500	1796	145 (8.1)		
Missing	884	278 (31.5)		

Supplementary Table 6. Effects of CD4 count before ART on loss to follow-up in HIV-infected patients who started ART between 2010 and 2015 in Guangxi, China

Variable	Number	Loss to follow-up	Person years	Loss to follow-up /100 person years (95% CI)	HR (95% CI)	P-value	AHR* (95% CI)	P-value
Total	49155	4507	129837.65	3.47(3.37-3.57)				
CD4 count before ART (cells/mm^3)								
<350	41554	3822	114372.74	3.34(3.24-3.45)	1.00		1.00	
350-499	4921	388	10118.95	3.83(3.45-4.22)	0.99(0.89-1.10)	0.835	1.01(0.90-1.12)	0.898
≥500	1796	155	3328.63	4.66(3.92-5.39)	1.14(0.97-1.34)	0.102	1.14(0.96-1.34)	0.126
Missing	884	142	2017.31	7.04(5.88-8.20)	1.96(1.66-2.32)	<0.001	1.89(1.60-2.24)	<0.001

* HR=hazard ratio; AHR=adjusted hazard ratio; covariates of the adjusted model included: age, sex, marital status, route of HIV infection, WHO clinic stage before ART, initial ART regiment, year initiated ART.

Supplementary Table 7. Effects of CD4 count before ART on medication cessation in HIV-infected patients who started ART between 2010 and 2015 in Guangxi, China

Variable	Number	Medication cessation	Person years	Medication cessation /100 person years (95% CI)	HR (95% CI)	P-value	AHR* (95% CI)	P-value
Total	49155	2396	129837.65	1.85(1.77-1.92)				
CD4 count before ART (cells/mm^3)								
<350	41554	1981	114372.74	1.73(1.66-1.81)	1.00		1.00	
350-499	4921	272	10118.95	2.69(2.37-3.01)	1.33(1.17-1.51)	<0.001	1.19(1.04-1.36)	0.009
≥500	1796	99	3328.63	2.97(2.39-3.56)	1.39(1.14-1.70)	0.001	1.21(0.99-1.49)	0.068
Missing	884	44	2017.31	2.18(1.54-2.83)	1.16(0.86-1.56)	0.341	1.08(0.80-1.46)	0.611

* HR=hazard ratio; AHR=adjusted hazard ratio; covariates of the adjusted model included: age, sex, marital status, route of HIV infection, WHO clinic stage before ART, initial ART regiment, year initiated ART.

Supplementary Table 8. Effects of CD4 count before ART on loss to follow-up in HIV-infected patients who started ART between 2010 and 2015 in Guangxi, China (Analytic sample does not include individuals with missing baseline CD4 counts)

Variable	Number	Loss to follow-up	Person years	Loss to follow-up /100 person years (95% CI)	HR (95% CI)	P-value	AHR* (95% CI)	P-value
Total	48271	4365	127820.33	3.41(3.31-3.52)				
CD4 count before ART (cells/mm^3)								
<350	41554	3822	114372.74	3.34(3.24-3.45)	1.00		1.00	
350-499	4921	388	10118.95	3.83(3.45-4.22)	0.99(0.89-1.10)	0.821	1.02(0.91-1.13)	0.777
≥500	1796	155	3328.63	4.66(3.92-5.39)	1.14(0.97-1.34)	0.105	1.15(0.97-1.35)	0.101

* HR=hazard ratio; AHR=adjusted hazard ratio; covariates of the adjusted model included: age, sex, marital status, route of HIV infection, WHO clinic stage before ART, initial ART regiment, year initiated ART.

Supplementary Table 9. Effects of CD4 count before ART on medication cessation in HIV-infected patients who started ART between 2010 and 2015 in Guangxi, China (Analytic sample does not include individuals with missing baseline CD4 counts)

Variable	Number	Medication cessation	Person years	Medication cessation /100 person years (95% CI)	HR (95% CI)	P-value	AHR* (95% CI)	P-value
Total	48271	2352	127820.33	1.84(1.77-1.91)				
CD4 count before ART (cells/mm^3)								
<350	41554	1981	114372.74	1.73(1.66-1.81)	1.00		1.00	
350-499	4921	272	10118.95	2.69(2.37-3.01)	1.33(1.17-1.51)	<0.01	1.19(1.04-1.36)	0.010
≥500	1796	99	3328.63	2.97(2.39-3.56)	1.39(1.14-1.70)	0.001	1.21(0.98-1.49)	0.071

* HR=hazard ratio; AHR=adjusted hazard ratio; covariates of the adjusted model included: age, sex, marital status, route of HIV infection, WHO clinic stage before ART, initial ART regiment, year initiated ART.
